# Engineering Photocarrier Redistributions in Graphene/III‐V Quantum Dot Mixed‐Dimensional Heterostructures for Radiative Recombination Enhancements

**DOI:** 10.1002/smll.202406197

**Published:** 2024-11-15

**Authors:** Rafael Jumar Chu, Quang Nhat Dang Lung, Tsimafei Laryn, Won Jun Choi, Daehwan Jung

**Affiliations:** ^1^ Center for Quantum Technology Korea Institute of Science and Technology Seoul 02792 South Korea; ^2^ Division of Nano and Information Technology KIST School at Korea National University of Science and Technology Seoul 02792 South Korea

**Keywords:** carrier dynamics, graphene, InAs, mixed‐dimensional, photoluminescence, quantum dot

## Abstract

Integration of graphene and quantum dots (QD) is a promising route to improved material and device functionalities. Underlying the improved properties are alterations in carrier dynamics within the graphene/QD heterostructure. In this study, it is shown that graphene functions as a carrier redistribution and supply channel when integrated with InAs QDs. Photoluminescence (PL) spectroscopy provides evidence that graphene modifies the redistribution, escape, and recombination dynamics of carriers in the InAs QD ensemble, which ultimately leads to enhanced radiative recombinations at all temperatures and excitation densities probed. It is also shown that the PL enhancement from the graphene/InAs QD heterostructure is greatest with a thin GaAs cap and at higher temperatures where devices operate. This study advances the understanding of graphene/QD heterostructures and can aid the design of mixed‐dimensional optoelectronic devices.

## Graphical abstract

Here, it is shown that graphene functions as a carrier redistribution and supply channel when integrated with III‐V quantum dots (QDs). Photoluminescence spectroscopy provides evidence that graphene modifies the redistribution, escape, and recombination dynamics of carriers in the InAs QD ensemble, which ultimately leads to enhanced radiative recombinations at all temperatures and excitation densities probed.

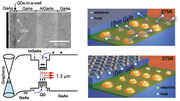

## 1 Introduction

Mixed‐dimensional heterostructures offer a pathway to obtaining properties that are otherwise unobtainable from individual materials separately. One can choose from a growing selection of 2D materials and mix them with 0D materials to obtain 2D‐0D mixed‐dimensional heterojunctions for photovoltaic, light sensing, and light emitting devices.^[^ ^1^, ^2^, ^3^ ^]^ The most mature 2D material is graphene, with its well‐known zero‐bandgap structure, ultrahigh carrier mobility, and thermal conductivity.^[^ ^4^, ^5^ ^]^ Integrating graphene on bulk III‐V semiconductors or other 2D materials is now routinely studied for hybrid solar cells, photodetectors, and LEDs.^[^ ^6^, ^7^, ^8^, ^9^, ^10^ ^]^ Due to the limited light absorption of graphene, colloidal QDs are often integrated onto graphene to either enhance its light collection ability or extend its response wavelength.^[^ ^11^, ^12^, ^13^, ^14^ ^]^ The QDs act as the absorber material that generates photocarriers, which then transfer to graphene and can be collected as photocurrent.^[^ ^15^, ^16^ ^]^ Because of the ultrahigh mobility of graphene and the long carrier lifetime of QDs, the photogenerated carriers that remain can recirculate many times between graphene and the QDs, leading to photoconductive gain.^[^ ^12^, ^17^ ^]^ This charge transfer, however, requires close proximity between graphene and the QDs.^[^ ^18^ ^]^

In the infrared regime, epitaxially grown InAs QDs are among the most well‐studied quantum emitters. By carefully controlling the QD dimensions and the barrier potentials around the QDs, the ground state (GS) emission can be tuned from 1.1–2.0 µm.^[^ ^19^, ^20^ ^]^ In particular, the 1.3 µm variety is well‐studied due to its importance in data communication.^[^ ^21^ ^]^ However, research on integrating epitaxial QDs and 2D materials is few and far between.^[^ ^17^, ^22^, ^23^, ^24^ ^]^ Wang et al. integrated graphene and InAs QDs for mode‐locked lasing applications.^[^ ^23^ ^]^ They demonstrated enhanced carrier transfer from graphene to QD across layers of InGaAs/GaAs. Hu et al. studied a QD/graphene phototransistor utilizing epitaxial InGaN QDs and demonstrated photodetection gain of up to 10^9^, which they attributed to carrier recirculation within graphene.^[^ ^17^ ^]^ Recently, our group has shown that by combining graphene and InAs QDs separated by a 10 nm GaAs interlayer, the photoluminescence (PL) from the InAs QDs can be enhanced up to 13 times by repairing the carrier imbalance in the InAs QDs.^[^ ^24^ ^]^ The graphene/InAs QD heterostructure deserves comprehensive optical analyses to understand its properties at a variety of conditions and the carrier dynamics that underlie its enhanced performance.

In this paper, we show that graphene acts as a carrier redistribution channel in a mixed‐dimensional graphene/QD heterostructure. Changes in PL spectral linewidths clearly indicate that graphene serves as an additional carrier supply route. Power‐dependent study also shows that graphene increases the hole concentration which changes the dominant quenching channel and increases the electron‐hole pair occupancy in the QD ensemble. These advantages disappear at low temperatures and with a thick GaAs cap, highlighting the fact that the benefits of graphene/QD integration are greatest with a 10 nm separation and at higher temperatures where devices operate. The insights gleamed in this study can assist in the design of mixed‐dimensional optoelectronic devices with improved material properties and device functionalities.

## 2 Results and Discussion

**Figure 1:**
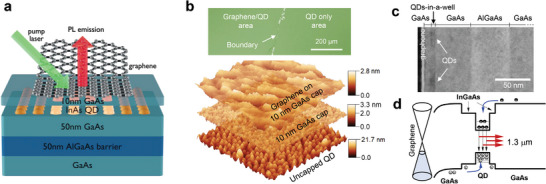
a) Schematic of the graphene/QD heterostructures with a 10 nm GaAs cap. b) Optical microscope image to show the edge of transferred graphene and atomic force microscope image stacks of uncapped QDs, 10‐nm GaAs‐capped QDs, and graphene‐covered surface. c) Transmission electron microscope image of the graphene/QD heterostructures with a 10 nm GaAs cap. d) Band alignment at the graphene/GaAs/QD interface.

**Figure** **1**a shows the schematic diagram of the graphene/InAs QD heterostructure studied here. All III‐V layers were grown on GaAs wafers by molecular beam epitaxy. The InAs QDs were grown in a dot‐in‐a‐well (DWELL) configuration and capped with GaAs with a thickness of either 10 or 50 nm (see Experimental Section). Graphene was then transferred via a modified wet transfer method (Figure S1, Supporting Information).^[^ ^24^ ^]^ After the transfer, the samples were immersed in acetone and then annealed at 400 °C for 30 min under N₂ gas to remove polymethyl methacrylate residues adhering to graphene, a critical step for achieving the hole doping concentration of graphene. Figure 1b shows an optical microscope image that captures the edge of the transferred graphene layer. 1 × 1 µm^2^ atomic force microscope (AFM) images show uncapped QD morphologies with an average QD density of 3 × 10^10^ cm^−2^. The sample with a 10 nm GaAs cap shows that the QDs are fully buried for graphene transfer. After transfer, the heterostructure surface also remains smooth, confirmed by the AFM measurement. The cross‐sectional transmission electron microscope image of the sample (Figure 1c) confirms the presence of a conformally transferred graphene, the absence of a native III‐V oxide, and a QD‐graphene distance of 10 nm as designed. Figure 1d shows the band alignment near the graphene‐QD heterojunction. This short distance is crucial for the fast hole transfer from graphene to QD.

Photoluminescence measurements were then conducted at various temperatures and excitation powers. All samples were loaded into a cryostat for PL measurements by applying the silver paste on the backside, and the graphene layer was electrically floating without any electrical connection. We define the PL enhancement factor as IPL_Gr/QD_/IPL_QD_, where IPL_Gr/QD_ is the integrated PL intensity (IPL) of the graphene‐covered region and IPL_QD_ is the IPL of the QD sample without graphene. Hereafter, measurements made on uncapped regions will be referred to as QD10 and QD50 for samples capped with 10 and 50 nm GaAs. Meanwhile, measurements made on graphene‐covered areas will be referred to as Gr/QD10 and Gr/QD50, respectively. At room temperature and ambient conditions, all measurements exhibit PL near 1.3 µm (Figure S2, Supporting Information).

### 2.1 Temperature‐Dependent Carrier Dynamics

**Figure 2:**
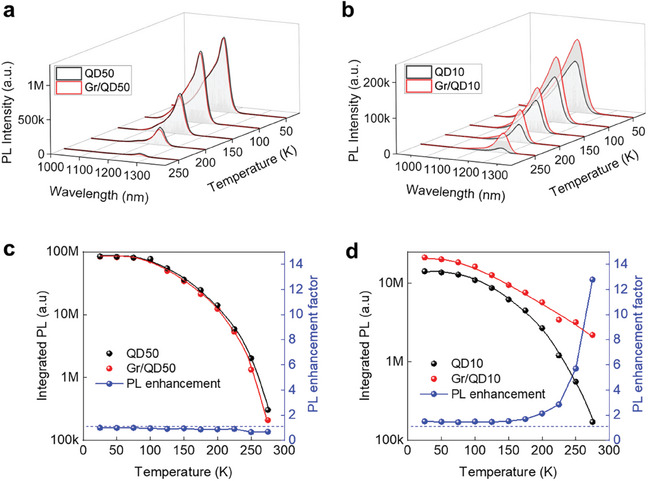
a,b) Temperature‐dependent photoluminescence spectra of (a) QD50 and Gr/QD50 and (b) QD10 and Gr/QD10. c,d) Integrated photoluminescence intensity and enhancement factor of (c) QD50 and Gr/QD50 and (d) QD10 and Gr/QD10.

**Figure** **2**a,b shows the temperature‐dependent photoluminescence spectra from the QD‐only and Gr/QD regions. At increasing temperatures, the peak energy shrinks faster than the Varshni model for bulk InAs, but no significant differences were observed among the different samples (Figure S3, Supporting Information). In the next sections, the IPL and full width at half maximum (FWHM) will be discussed separately.

Figure 2c shows the IPL and PL enhancement factor of QD50 and Gr/QD50 across the temperature range studied. Evidently, the 50 nm separation between graphene and the QDs is too large to have any appreciable effect on the PL spectra. A slight decrease is observed which could be due to graphene's parasitic absorption of the incoming pump laser and of the exiting PL. On the other hand, Gr/QD10 exhibits enhancement in the entire temperature range studied (Figure 2d). The PL enhancement factor is 1.4–1.5 from 25–150 K and increases rapidly to 13 at 275 K. As argued previously, graphene positioned close to QDs repairs the different electron and hole carrier densities in the QD and leads to enhancement in PL intensity.^[^ ^24^ ^]^ This finding indicates that the advantages of integrating graphene and InAs QD are most significant for vertically compact structures (i.e., with a thin 10 nm cap), at temperatures where devices are typically operated, and at temperatures where heating becomes a performance limitation. We previously studied the relationship between PL enhancement factor and GaAs cap thickness and the graphene/QD interaction is stronger with shorter GaAs cap thickness.^[^ ^24^ ^]^ Also, the PL enhancement is not associated with carrier diffusion length, but with energy band alignment and bending at the graphene/QD interfaces. Previous studies on colloidal QD/graphene hybrid photodetectors revealed that one type of charge carriers transfers from QDs to graphene and this carrier trapping generates effective photoconductive gain.^[^ ^25^ ^]^ However, our graphene layer is floating without any external bias and extra holes are transferred from QDs to the heterostructure for improved radiative recombination rates. Therefore, it should also be interesting if one can measure light‐induced voltages or tune the photocarrier transfers between graphene and III‐V QDs by applying external bias, which will be investigated for future work.

### 2.2 Carrier Redistribution

We then studied the temperature dependence of the PL spectral linewidth and shape, which provide detailed information about the occupancy of energy states within the QD ensemble.^[^ ^26^, ^27^ ^]^ In our experiments, the laser beam is large enough to directly pump an area corresponding to ≈10^9^ QDs. Photocarriers generated throughout the structure can further diffuse laterally into a much larger area with more QDs. Thus, for macro‐PL spectroscopy, a GS peak is actually a convolution of narrow peaks obtained from a QD population of different sizes. Corollary to this, an asymmetric change in PL shape should correspond to a redistribution of carriers across the dimensionally broad QD population.

#### 2.2.1 50 nm Cap

**Figure 3:**
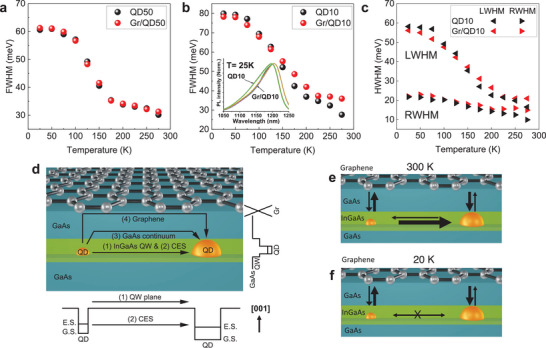
Temperature‐dependence of the full‐width at half maximum for QD‐only and graphene‐capped samples with a GaAs cap thickness of a) 50 nm and b) 10 nm. Inset in (b) shows five different photoluminescence spectra measured at 25 K. c) Left‐width and right‐width at half maximum values for the 10 nm GaAs cap sample. d) Schematic illustration of the carrier transfer channels between quantum dots. e) At 300 K, all carrier transfer channels are potentially available. f) At 20 K, most of the channels are not available; however, carrier transfer via graphene is still possible.

First, we will look at QD50 which represents the typical structure where the QD layer is capped with a sufficiently thick GaAs layer. The FWHM of QD50 exhibits an S‐shape temperature‐dependence (**Figure** **3**a), commonly attributed to the thermally‐assisted carrier redistribution in DWELL structures.^[^ ^28^, ^29^ ^]^ Upon excitation with a pump laser, carriers will be captured randomly across the QD ensemble. At low temperatures, photocarriers that are captured inside these initial QDs will tend to remain inside the same QDs due to the low thermal energy and subsequently recombine radiatively. Hence, a larger distribution of QD sizes remains emissive, leading to a larger FWHM.

At higher temperatures, the photocarriers can be thermally excited and redistributed among QDs with different sizes. Figure 3d illustrates the various transfer channels available for a carrier to transfer to another QD. Carriers can either be excited 1) into the quantum well (QW) plane on which the carriers are free to diffuse laterally, 2) via coupled excited states (CES) among QDs, among which there is sufficient wave function overlap for a carrier to tunnel from one QD to the next, or 3) into the GaAs continuum within which the carrier can move in three dimensions.^[^ ^26^, ^30^, ^31^ ^]^ In any of these cases, an excited carrier can be captured by a defect state which quenches the PL intensity. Carriers will generally redistribute from smaller QDs (higher energy) to larger QDs (lower energy), since bigger QDs have a larger capture cross‐section and larger band offsets with the barrier.^[^ ^32^ ^]^ Capping with graphene does not lead to any changes in the temperature‐dependence of FWHM since 50 nm is simply too far to produce any effect on QD emission mechanism.

#### 2.2.2 10 nm Cap

When the GaAs cap thickness is reduced to 10 nm, the FWHM values still exhibit the typical S‐shape curve, but additionally, a graphene effect clearly emerges (Figure 3b). To acquire statistical FWHM data, we also measured multiple spots at the low and high temperatures, and highly precise FWHM values were obtained from the samples (More details are provided in Figure S4, Supporting Information). Comparing QD10 and Gr/QD10 shows a crossover point at 125–150 K, where Gr/QD10 has larger (smaller) FWHM values above (below) this point. Another set of temperature‐dependent PL measurements from a different Gr/QD10 sample with a newly transferred graphene also revealed a consistent result as Figure 3b but with more drastic differences in the FWHM values (See Figure S5, Supporting Information). To understand the origin of this crossover, we plotted the left width at half‐maximum (LWHM) and right width at half‐maximum (RWHM) values of the spectra, as shown in Figure 3c. In our analysis, a broadened RWHM is interpreted as redistribution into larger QDs (lower GS energy), while a broadened LWHM as redistribution into smaller QDs (higher GS energy). The RWHM barely changes across the entire temperature range for both QD10 and Gr/QD10. This is attributed to the big dots having larger capture cross sections and larger band offsets with the barrier levels which then reduces the carrier escape probability. The LWHM exhibits the S‐shaped trend for both QD10 and Gr/QD10. This trend is consistent with the idea that there is larger occupancy within small dots at low temperatures which contributes to the PL emission. As the temperature increases, the LWHM drops significantly because the escape from small QDs is more significant.

We then look at the effect of graphene on the LWHM and RWHM values. The high temperature (*T* > 150K) regime shows that graphene enhances the emission from both big dots and small dots as evidenced by the broadening of both RWHM and LWHM for Gr/QD10. In the latter sections, we will see that graphene increases the electron‐hole occupancy in the QDs, and so this increased carrier population also increases the emission from both big and small dots. This results in the broadening of both the long and short wavelength tails. Meanwhile, the low‐temperature regime (*T* < 125 K) shows that graphene leads to the narrowing of the LWHM. This suggests that carriers are being redistributed away from the small QDs. We believe that this is because graphene opens a carrier channel that allows carrier redistribution even at low temperatures as illustrated in Figure 3e,f. Electronic coupling should be possible for a distance of 10 nm. In vertically strain‐coupled QDs, even a 10‐nm GaAs spacer is enough to electronically couple the QDs in adjacent layers.^[^ ^33^ ^]^ Recent QD lasers take advantage of a p‐doped layer placed 10–15 nm away from the QDs to supply holes.^[^ ^34^ ^]^ In QD injection lasers, a QW placed near a QD can act as a carrier reservoir to enhance the optical performance of QDs.^[^ ^35^ ^]^ In an analogous manner, graphene placed 10 nm from the QD layer functions as a carrier supply and redistribution channel.

### 2.3 Thermal Activation Channels

**Figure 4:**
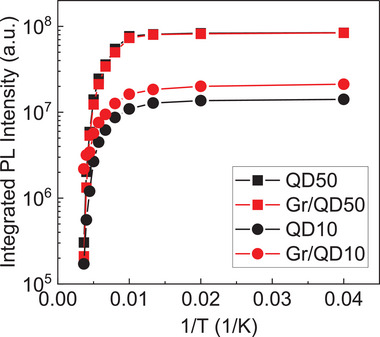
Arrhenius curve of the integrated PL intensity of all samples.

**Figure** **4** shows the Arrhenius plot of the IPL. All samples exhibit typical quenching behavior at increasing temperatures. Upon gaining thermal energy, the carriers are first thermally excited from the QD GS to either a higher energy state in the QD, QW, or barrier layer. Through this energy state, the carriers can either tunnel to a neighboring QD via a coupled energy state, diffuse, and be re‐captured by another QD, or be captured by a nearby defect state that quenches the PL. These are represented by paths 1–3 in Figure 3d. By fitting the IPL data, one can calculate the thermal activation energy of the QD ensemble and determine the dominant thermal escape channels of the carriers. Here we fitted the data with the following Equation:

(1) $$\mathrm{IPL}\;\left(T\right)=\frac{1}{1+\sum_{n}A_{n}\exp\left(-\;\frac{E_{\mathrm{an}}}{\mathrm{kT}}\right)}$$

where E_an_ are thermal activation energies and A_n_ are fitting parameters. We found that our data can be fitted well with 2 activation energies, E_a1_ and E_a2_, which are summarized in **Table** **1**.

**Table 1:** Summary of activation energies.

	E_a1_ [meV]	E_a2_ [meV]
QD50	314	56
Gr/QD50	345	60
QD10	180	33
Gr/QD10	65	15

#### 2.3.1 50 nm Cap

For Gr/QD50, there is a small increase in the activation energies compared to QD50. The larger E_a1_ is associated with the escape of carriers to the barrier state, either laterally to the QW or vertically to the GaAs barriers. This value matches the energy difference between the GS (0.95 eV) level and the InGaAs QW (1.265 eV), suggesting the escape of both electrons and holes to the InGaAs QW plane. Meanwhile, the smaller E_a2_ of ≈60 meV is close to the difference between the GS and excited state (ES) levels. The presence of graphene increases both E_a1_ and E_a2_ by some amount, but the values remain in a similar range, suggesting that graphene does not alter the thermal escape mechanism significantly when the distance is 50 nm.

#### 2.3.2 10 nm Cap

For QD10 and Gr/QD10, the activation energies varied significantly. First, the lower activation energies of QD10 compared to QD50 indicate that a thin cap alters the carrier escape mechanism. For QD10, the obtained value of 180 meV is 38% of the GS and GaAs bandgap difference, which is consistent with the escape of holes to the continuum.^[^ ^36^ ^]^ Meanwhile, 33 meV is consistent with the capture of excitons by defects in GaAs.^[^ ^37^ ^]^ Upon the integration of graphene, both E_a1_ and E_a2_ are altered to a different value range. We believe that this stems from the abundance of carriers in the Gr/QD10 system, which leads to two possible effects. First, the extra supply of holes from the graphene/QD interface helps replace the holes escaping into the GaAs continuum. Second, the extra carriers could saturate the defect states. Hence, carrier escape and defect capture cease to be the predominant quenching mechanisms. In our transfer process, the transferred graphene has a hole sheet concentration of 8.5 × 10^12^ cm⁻^2^ as measured by the Hall effect on calibration samples; this roughly translates to 285 holes/QD. This supply of extra carriers, coupled with the idea that carriers can recirculate between graphene and QDs should be sufficient to replenish the escaping holes and saturate the quenching states near or within the 10‐nm GaAs cap. The larger energy of 65 meV for Gr/QD10 is very close to the energy separation between ES and GS, suggesting carrier escape into the excited states and quenching into coupled defect states. Meanwhile, the lower energy of 15 meV is close to the energy separation of states in the tightly‐packed valence band. To summarize, in QD10, the predominant quenching mechanism is related to the escape of holes to the GaAs barrier and their subsequent capture into GaAs‐related defect states. By repairing the imbalance of carriers and possibly saturating the defects, graphene integration alters the predominant escape mechanism to that related to excited states and hole escape via the tightly‐packed valence states.

### 2.4 Excitation‐Dependent Photoluminescence

To further explore the carrier dynamics in the graphene/QD heterostructure, we performed excitation‐dependent PL spectroscopy at 25 K and 275 K. The increase in IPL with excitation power I_0_ generally follows the power law,

(2) $$\mathrm{IPL}=\eta {I_{0}}^{\alpha }$$

where α characterizes the recombination processes occurring in the QD system and η is a fitting parameter. In log‐log scale, the slope α typically assumes values between 1 and 2.^[^ ^36^, ^38^ ^]^ There are three factors that can affect the linearity of the IPL with excitation, all of which are related to how carriers escape from the QD ground state to a barrier state.^[^ ^36^ ^]^ (1) The linearity is related to the excitonic nature of the carriers. A linear trend (α = 1) points to the excitonic escape of carriers, while a quadratic trend (α = 2) points to a fully independent escape of electrons and holes. (2) Across a larger range of excitation powers, the linearity is related to the occupancy of carriers within the QD, where we expect a shift from superlinearity to linearity when the average number of electron‐hole (e‐h) pairs/QD increases. (3) The barrier height can affect the linearity of the trend, with smaller barrier heights leading to superlinearity due to increased carrier escape. 25 K was chosen for low‐temperature measurement because it was the lowest temperature that our cryostat could control steadily. Also, 25 K is low enough to observe excitonic behaviors of e‐h pairs generated in the novel graphene/QD heterostructures. On the high‐temperature regime, we chose 275 K for several reasons. First, 275 K is high enough to measure free carrier recombinations over excitonic recombinations. Also, the sample emitted stronger PL intensities at 275 K than 300 K when pumped at a low excitation laser power (<1 mW).

#### 2.4.1 Excitation‐Dependence at 25 K

**Figure 5:**
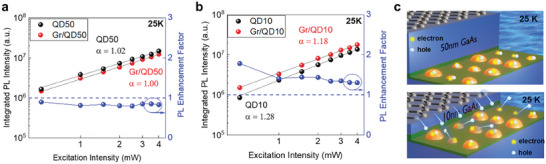
a,b) Integrated PL intensity at 25K and PL enhancement at different powers for samples with (a) 50 nm and (b) 10 nm GaAs cap. c) Schematic illustrations of carrier occupancies at 25 K for Gr/QD50 and Gr/QD10.

We then focus on the excitation‐dependence of the IPL (**Figure** **5**a,b). Figure S4 (Supporting Information) shows the PL spectra taken under low excitation power levels for all samples at 25K. The excitation powers used were low enough that the PL shape (peak wavelength and FWHM) remained unaltered across the values used in our experiment. For QD50 and Gr/QD50 (Figure 5a), the trend shows a linear dependence at 25K, consistent with either excitonic recombination or a high carrier occupancy in the QDs. The very close α values (**Table** **2**) for both samples suggest that the inclusion of graphene does not modify the carrier dynamics in QDs with a 50 nm GaAs cap. The PL enhancement factor shows no substantial variation in response to different incident powers but remains below 1, consistent with the previous section. The reduced PL intensity from Gr/QD50 compared to QD50 is due to the photon absorption of the graphene layer. Monolayer graphene is known to have ≈2% optical absorption in the visible and near‐infrared wavelength regimes. Therefore, the graphene layer first reduces the excitation laser power, which leads to a weaker PL emission. Then, the emitted photons from Gr/QD50 are absorbed one more time by the graphene on the surface.

**Table 2:** Summary of α values at 25 and 275 K for all samples.

	25 K	275 K
QD50	1.02	1.29
Gr/QD50	1.00	1.23
QD10	1.28	1.38
Gr/QD10	1.18	0.99

On the other hand, both QD10 and Gr/QD10 exhibit a small superlinear dependence at 25 K (Figure 5b). The lower barrier height in the thin 10‐nm GaAs cap facilitates carrier escape even at 25 K and leads to superlinearity. In the previous section, we identified hole escape as the major quenching channel for QD10. This leads to an imbalance in the electron and hole densities which manifests as an increase in α value from 1 to 1.28. This imbalance in electron and hole densities can be counterbalanced by incorporating graphene in proximity to the QD active layer. As a result of the increased hole concentration, the integration of graphene leads to a decrease in the α value from 1.28 to 1.18. The increase in carrier occupancy subsequently results in the enhancement in PL intensity. The PL enhancement factor (Figure 5b) reaches 1.8 at the lowest excitation power used. At higher powers, the PL enhancement factor saturates at ≈1.3, possibly due to the saturation of holes and defects. The carrier occupancies at 25 K are schematically illustrated in Figure 5c. As mentioned earlier, the 50 nm GaAs cap is thick enough to confine the generated carriers inside the QDs. Therefore, both Gr/QD50 and QD50 samples possess one e‐h pair per QD at 25 K. In the case of the 10 nm GaAs cap, QDs do not keep hole carriers for every QD even at 25 K as evidenced by the excitation‐dependent PL result and α values greater than 1. Then, the graphene supplies more hole carriers efficiently to the QDs in the Gr/QD10 heterostructure (Figure 5c bottom image) and increases e‐h pair occupancies as proved by the α value change from 1.28 to 1.18.

#### 2.4.2 Excitation‐Dependency at 275 K

**Figure 6:**
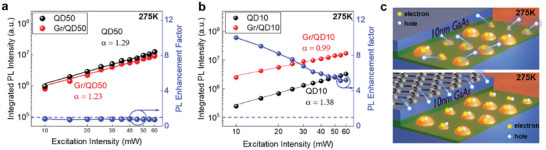
Integrated PL intensity at 275 K and PL enhancement at different powers for samples with a) 50 nm and b) 10 nm GaAs cap. c) Schematic illustrations of carrier occupancies at 275 K for Gr/QD50 and Gr/QD10.

Excitation‐dependent PL was also performed at 275 K at low excitation densities. Figure S6 (Supporting Information) shows the PL spectra of all samples, exhibiting no change in peak wavelength and FWHM across the powers used. However, compared to 25 K, the PL intensities at 275 K is quenched significantly as the thermal escape rate becomes very high. In addition, electrons and holes can no longer remain as bound excitons at this temperature. It is therefore anticipated that all samples will exhibit a superlinear dependence with excitation power. Indeed, superlinear trends were observed for both QD50 (α = 1.29) and QD10 (α = 1.38), as seen in **Figure** **6**a,b. This superlinearity is interpreted to both mean the independent escape of the two carrier types and therefore, the low carrier occupancy (e‐h pair/QD < 1). The larger α of QD10 is believed to be due to its smaller effective barrier height which increases the carrier escape rate.

For Gr/QD50, we obtained an α value of 1.23, which suggests that graphene integration does not significantly alter the recombination mechanism in the QD layer when graphene is placed at a 50 nm distance. However, when the distance of graphene was lowered to 10 nm, the dependence switched from superlinearity (QD10, α = 1.38) to linearity (Gr/QD10, α = 0.99). As with the case of Gr/QD10 at 275 K, this lower α is due to the increase in carrier occupancy to 1 or more e‐h pair/QD, suggesting that graphene increases the e‐h pair occupancy in the QDs. The difference in carrier occupancy can also be deduced from the PL shape. In QD10, only the GS emission can be observed, but in Gr/QD10, the 1st ES level begins to appear and gradually increases in intensity as the excitation power is increased. Furthermore, the α value was only lowered to 1.18 at 25 K, but at 275 K, it was lowered to 0.99, suggesting that the increase in carrier occupancy is more pronounced at 275 K. The carrier occupancies at 275 K are schematically illustrated in Figure 6c. Similarly, the temperature‐dependent PL enhancement factor also exhibits its highest value at 275 K. The QD10 sample at 275K suffers from thermal escapes of holes, leaving QDs unpopulated by e‐h pairs. However, as illustrated in the bottom image of Figure 6c, the graphene layer quickly supplies the hole carriers to the QDs in Gr/QD10 and increases e‐h pair occupancies in the QDs. Thus, these results underscore our finding that the effect of graphene integration is most pronounced at higher temperatures where devices operate. Also, further studies on the temperature dependency beyond 300K should be interesting to unveil the carrier redistribution at elevated temperatures as previous studies on other 2D materials such as WSe2 and MoS2 showed enhanced photoemission.^[^ ^39^, ^40^ ^]^

The excitation‐dependent PL enhancement factor for Gr/QD10 is much higher than that of Gr/QD50, which also further highlights the importance of the proximity of graphene. There is, however, a decrease in PL enhancement factor with increasing excitation power. This happens because, at high excitation densities, the rate at which photogenerated holes are supplied from the GaAs barriers to the QDs is greater than the rate at which graphene re‐supplies holes into the QDs.

## 3 Conclusion

Graphene/QD heterostructures exhibit enhanced photoluminescence intensities due to the supply of holes by energy band bending and carrier recirculation. In this study, we show evidence that graphene acts as a channel for photocarrier supply and redistribution. Temperature‐dependent and excitation‐dependent PL analysis show that graphene alters the carrier escape, redistribution, and recombination mechanisms in QDs when the distance is 10 nm. We observed that graphene enabled carrier transfer even at low temperatures where carriers are normally trapped without graphene. Calculations of activation energies reveal that graphene modified the dominant escape channels – where the PL was originally quenched by the escape of holes to the barrier, the increase in carriers in the graphene sample effectively suppressed this escape channel. Excitation‐dependent PL further supports our findings by showing changes in excitation dependency from superlinear to linear with the presence of graphene. These findings underscore the significance of graphene in modulating the carrier dynamics and dependence characteristics in quantum dot structures, offering valuable insights for tailoring optoelectronic devices with desired performance attributes.

## 4 Experimental Section

### Epitaxial Growth of InAs QDs

The self‐assembled InAs QDs were grown via the Stranski–Krastanov mode on semi‐insulating GaAs (001) substrates in a Veeco Gen930 molecular beam epitaxy chamber.^[^ ^20^, ^24^ ^]^ A 50 nm GaAs buffer layer was first grown followed by a 50 nm Al_0.4_Ga_0.6_As blocking layer and a 50 nm GaAs layer. The active region was then grown in a dot‐in‐well configuration consisting of InAs QDs buried within an In_0.15_Ga_0.85_As quantum well. The nominal thickness of the deposited InAs and the growth temperature were tuned to obtain peak emission near 1.3 µm at room temperature. A 2.5 nm cold GaAs cap was grown, and finally, the sample was capped with high‐temperature GaAs with a thickness of either 10 or 50 nm. Calibration samples were grown with uncapped QDs and calculated an average QD density of 3 × 10^10^ cm^−2^. AFM and TEM observations show that the QDs have a lateral size of 10–20 nm, average center‐to‐center distance of ≈60 nm, and edge‐to‐edge distance of 20–30 nm. The 10‐nm‐thick GaAs surface has an average root‐mean‐square (RMS) roughness value of 0.34 nm, indicating that the QDs were buried well. This RMS value is low enough to ensure conformal contact between the GaAs surface and graphene, which is ideal for the uninterrupted carrier transfer between the two materials.

### Graphene Transfer Process

The as‐grown quantum dot samples were first immersed in a solution of HCl and H2O (1:5) for 1 min to remove the native oxide. Subsequently, commercial chemical vapor deposition‐grown graphene purchased from Graphene Supermarket was transferred onto the samples by wet transfer following the steps described in the Supporting Information.

### Photoluminescence Measurements

Photoluminescence measurements were conducted using a power‐adjustable 532 nm laser (beam diameter = 1.9 mm) modulated by an optical chopper. Signals were collected using a monochromator and InGaAs photodetector connected to a lock‐in amplifier. Low‐temperature measurements were obtained after loading the samples in a cryostat chamber cooled by a He cryo compressor.

## Conflict of Interest

The authors declare no conflict of interest.

## Supporting information



Supporting Information

## Data Availability

The data that support the findings of this study are available from the corresponding author upon reasonable request.
