# Supplementary material for: Engineering Photocarrier Redistributions in Graphene/III‐V Quantum Dot Mixed‐Dimensional Heterostructures for Radiative Recombination Enhancements
Source: Small. 2024 Nov 15;21(1):2406197. doi: 10.1002/smll.202406197 (PMC11707559; doi:10.1002/smll.202406197)
Supplement: Supplementary file 1 — Supporting Information [file SMLL-21-2406197-s001.docx]

Supporting Information

Engineering Photocarrier Redistributions in Graphene/III-V Quantum Dot Mixed- Dimensional Heterostructures for Radiative Recombination Enhancements

*Rafael Jumar Chu^1,2,‡^, Quang Nhat Dang Lung^1^ ^‡^, Tsimafei Laryn^1,2^, Won Jun Choi^1^, and Daehwan Jung^1,2^,**

1 Center for Quantum Technology, Korea Institute of Science and Technology, Seoul, 02792, South Korea

2Division of Nano and Information Technology, KIST School at Korea National University of Science and Technology, Seoul, 02792, South Korea

^‡^Equally contributed

*Email: [daehwan.jung@kist.re.kr](mailto:daehwan.jung@kist.re.kr) #Corresponding author

S1. Graphene transfer process


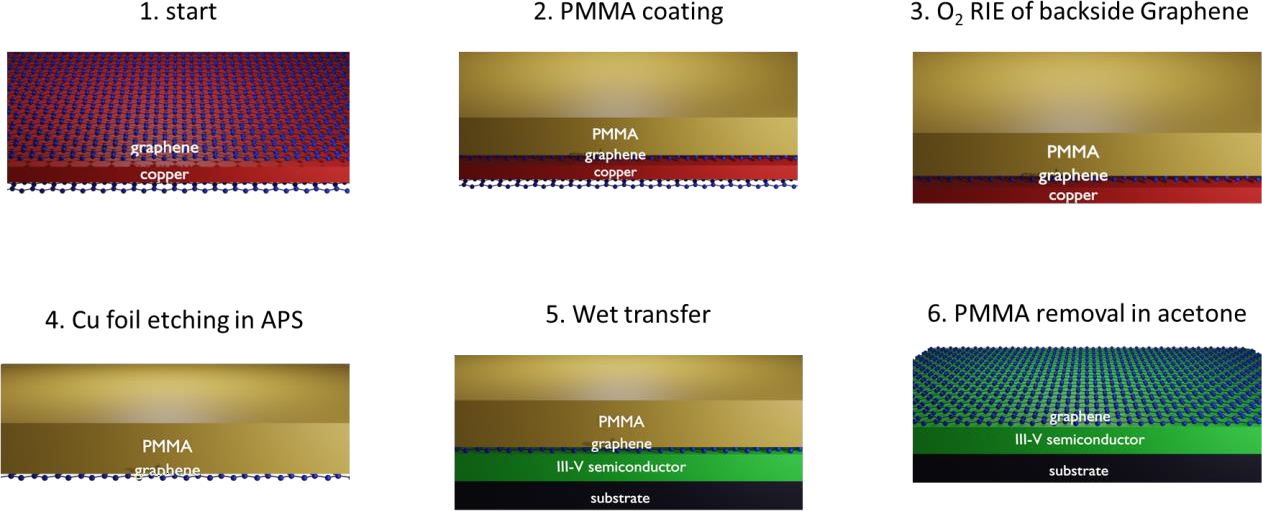


Figure S1. A modified graphene wet-transfer process onto III-V quantum dot sample.

Graphene was purchased from *Graphene Supermarket*. The CVD-grown graphene is present in both sides of the Cu foil. Polymethyl methacrylate (PMMA) was deposited on one side of the sample as a supporting layer. The exposed graphene was etched by O2-RIE. Then, the Cu foil was etched in an Ammonium persulfate (APS) solution. The released PMMA/Graphene film was rinsed in HCl:H2O2:H2O (1:1:20) and DI water to remove the metal residues. Immediately preceding the graphene transfer, the QD sample was first dipped in HCl:H2O (5:1) for 1 min to remove any native oxide that has formed after MBE growth. The

sample was then dried and baked on a hot plate, first at 80°C and then at 180°C, to enhance the graphene/III- V adhesion. Subsequently, it was immersed in acetone, then annealed at 400°C for 30 minutes under N2 gas to further remove PMMA residues adhering to graphene. This annealing step is crucial for tuning the

hole doping concentration of graphene, as thoroughly discussed in our previous work.^[1]^


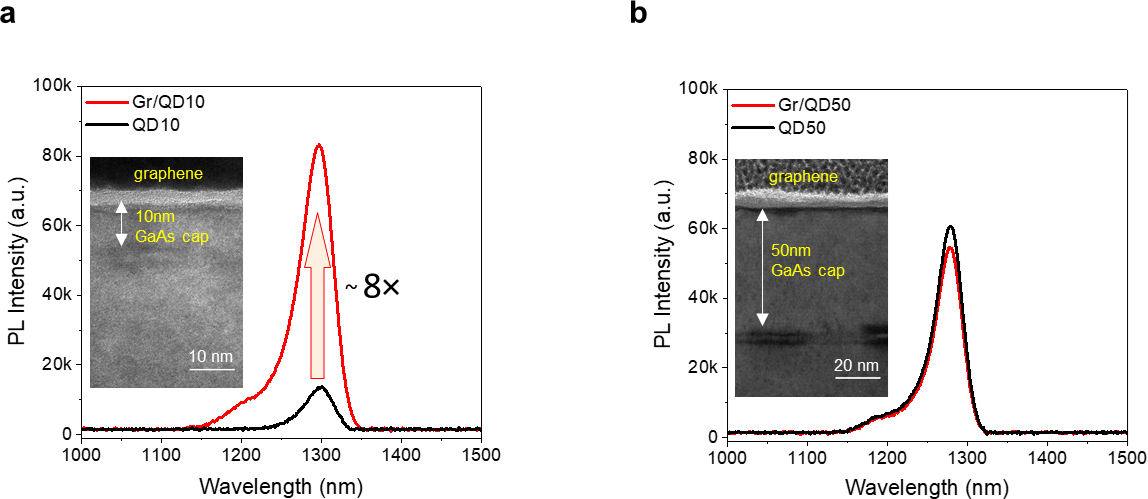


Figure S2. Photoluminescence spectra from the samples were collected at room temperature and ambient conditions.

# Photoluminescence spectra from the samples were collected at room temperature and ambient conditions. All measurements exhibit a peak near 1.3 μm. Upon graphene integration, Gr/QD10 shows an 8× enhancement in integrated PL intensity, while Gr/QD50 shows 0.9. Insets show transmission electron microscope images of both samples. The bright layer near the surface is graphene.


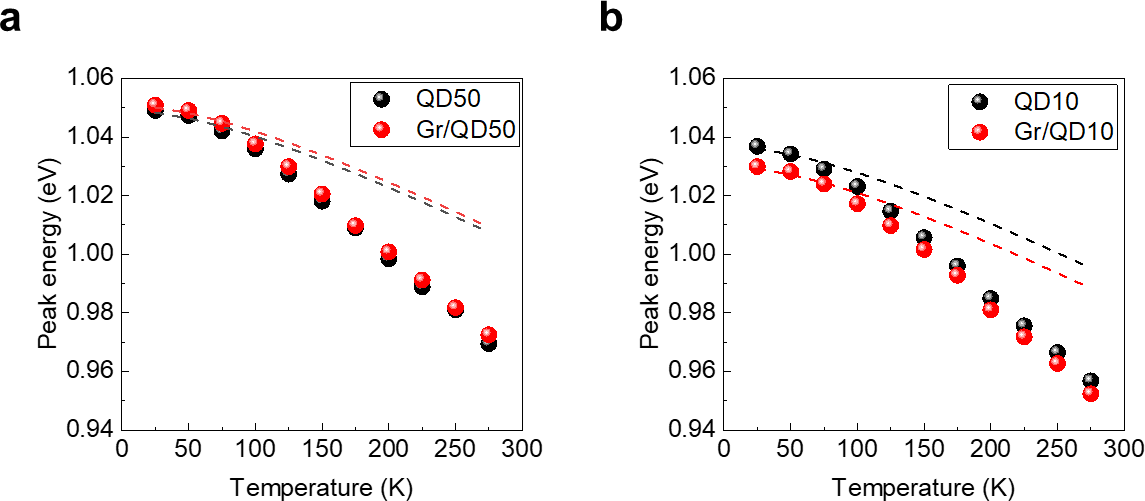


Figure S3. Temperature-dependence of the photoluminescence peak energy.

The temperature-dependence of the peak energy is plotted above. All samples exhibit a monotonic decrease in peak energy with temperature increase, which roughly corresponds to the well-known bandgap shrinkage of bulk semiconductors. The QD ground state energy shrinks faster than the Varshni model fitted using bulk InAs parameters (Varshni parameter =0.25 meV/K2 and Debye temperature =180). This has been ascribed to the thermal redistribution of carriers which favors QDs with lower GS energies.

The presence of graphene (Gr/QD50) does not lead to significant changes in the rate of bandgap shrinkage compared to QD50. However, Gr/QD10 is redshifted compared to QD10. We measured multiple spots and confirmed that the redshift is not due to QD growth variations. This redshift is due to enhanced carrier redistribution from smaller QDs to larger QDs with smaller GS transition energies.


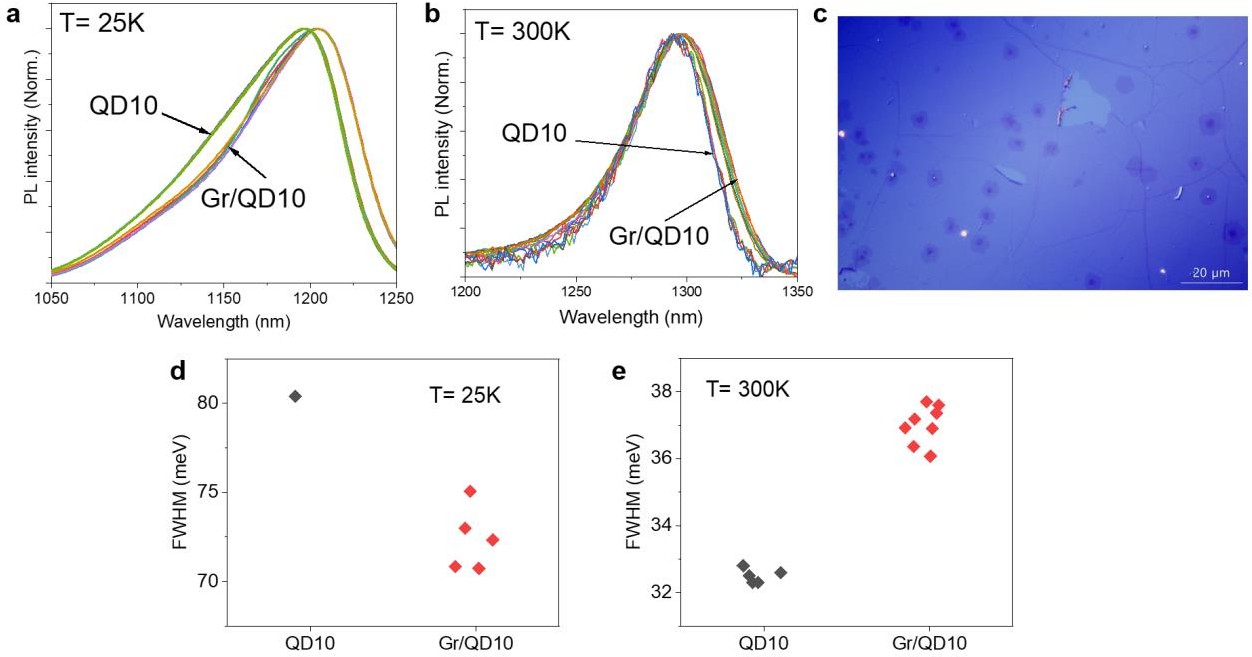


Figure S4. Photoluminescence spectrum from QD10 and Gr/QD10 samples at (a) 25K and (b) 300K with more than five different scans per temperature. (c) Optical microscope image to show grain boundaries, patches, and bilayers in the transferred graphene. FWHM values (a) at 25K and (b) 300K.

Figure S4a and S4b show that QD10 has very consistent PL data while Gr/QD10 shows slight variations both at 25K and 300K. Each figure shows more than five different PL scans. We could not collect an equal number of data points at other intermediate temperatures between 50K and 275K because T-dependent PL measurement is very time consuming, taking about 8 hours due to sample cooling and temperature stabilization at each step. The variations in Gr/QD10 should be caused by imperfect graphene conditions as seen in Figure S4c. We observed some grain boundaries and macro-sized patches, which are typical characteristics of CVD-grown graphene. Figure S4d and S4e summarize the FWHM values, which support our claim in Figure 3 well in the main text.


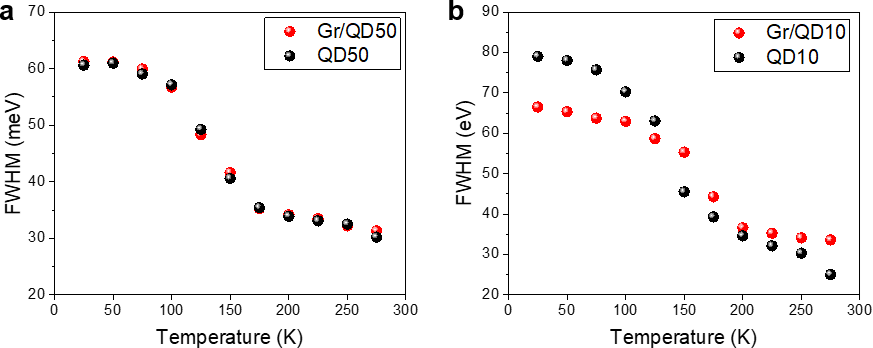


Figure S5. Temperature-dependent photoluminescence spectrum from another set of (a) Gr/QD50 and QD50 and (b) Gr/QD10 and QD10 samples.

We also performed another set of T-dependent PL experiments to provide more statistical data and to support our claim in the paper. Figure S5 above presents another set of temperature-dependent experiments, but from different spots of the sample samples. Still, Gr/QD50 and QD50 have almost identical FWHM evolutions over the measured temperatures while Gr/QD10 and QD10 reveal FWHM crossover point around 150K, which is very consistent to the result in the main manuscript.


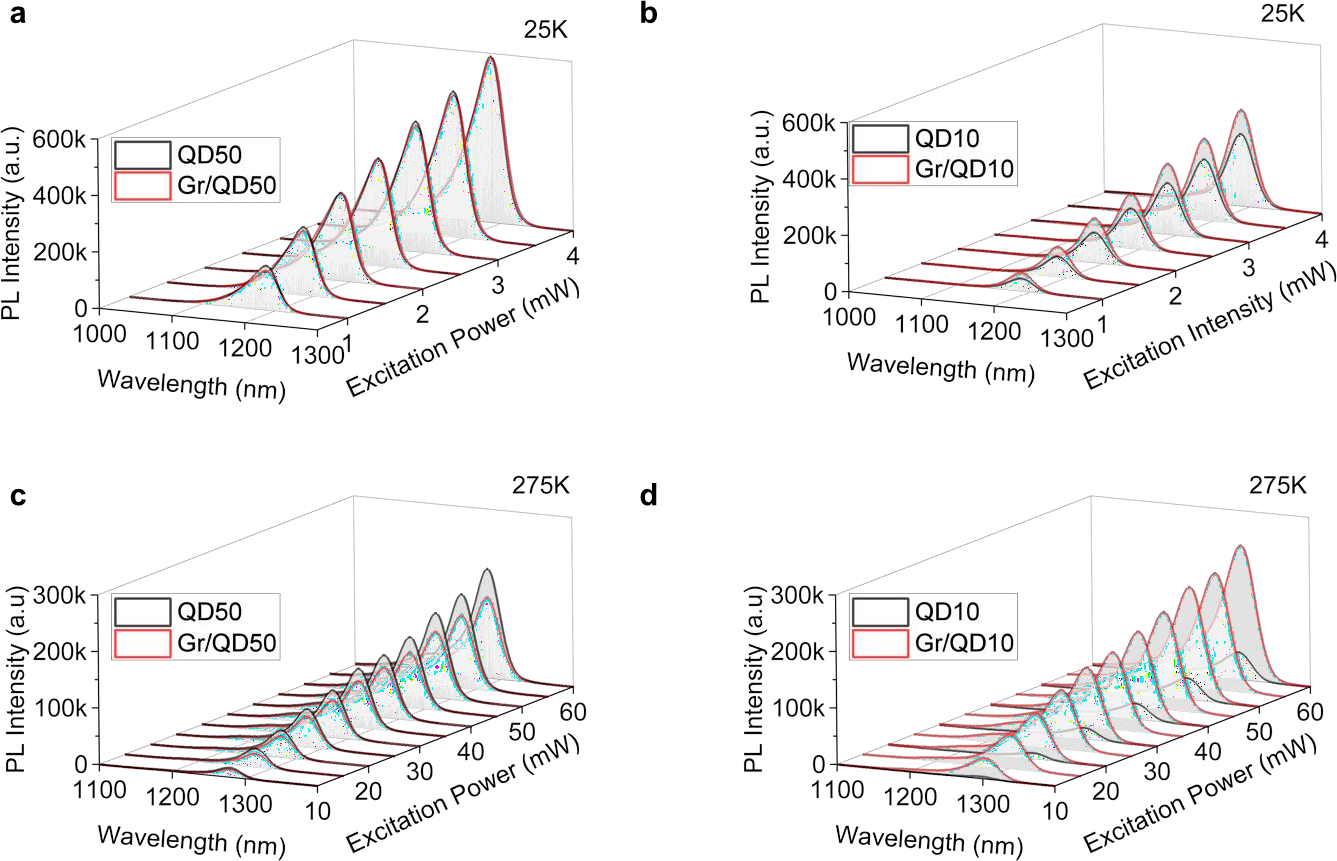


Figure S6. Excitation-dependent photoluminescence spectra from QD50, Gr/QD50, QD10, and Gr/QD10 measured at (a-b) 25 K and (c-d) 275 K.

References:

1. Q. N. D. Lung, R. J. Chu, Y. Kim, T. Laryn, M. A. Madarang, O. Kovalchuk, Y.‐W. Song, I.‐H. Lee, C. Choi, W. J. Choi, D. Jung, Nano Lett. **2023**, 23, 3344.
